# Diagnosis and management of neutropenia in adults: Expert guidance

**DOI:** 10.1111/bjh.70640

**Published:** 2026-06-29

**Authors:** Karl Welte, Cornelia Zeidler, Julia Skokowa, Sabine Mellor‐Heineke, Audrey Anna Bolyard, David Dale

**Affiliations:** ^1^ Department of Pediatric Hematology and Oncology University Children's Hospital Tuebingen Tuebingen Germany; ^2^ Department of Oncology, Hematology, Clinical Immunology, and Rheumatology University Hospital Tuebingen Tuebingen Germany; ^3^ Medical School Hannover Hannover Germany; ^4^ Department of Medicine University of Washington Seattle Washington USA

**Keywords:** autoimmune neutropenia, clonal haematopoiesis, drug‐induced neutropenia, febrile neutropenia, germline mutations, leukaemia, management of neutropenia, neutropenia, somatic mutations

## Abstract

Neutropenia in general is defined as a blood neutrophil count of less than 1.5 × 10^9^/L, in severe neutropenia counts drop to less than 0.5 × 10^9^/L, but the definition of neutropenia varies according to the patient's ethnic origin and age. For Caucasian adults, the absolute neutrophil count (ANC) threshold of 1.8 × 10^9^/L is adopted for the definition of neutropenia according to the World Health Organization (see Fioredda et al. (1)). It can be inherited or acquired, and it is an uncommon haematological finding in adult outpatient clinics. Neutropenia can result from decreased production of neutrophil precursors in the bone marrow, as in the case of severe congenital neutropenia, or from increased utilization of neutrophils in some bacterial infections, or accelerated destruction as is the case with drug‐induced neutropenia, viral infections and autoimmune neutropenia. Severe chronic neutropenia increases susceptibility to bacterial or fungal infections. Treating severe chronic neutropenia patients with granulocyte colony‐stimulating factor (G‐CSF) can increase neutrophil counts for most types of neutropenia. This article will provide guidance on diagnosing and managing severe chronic neutropenia in adult patients and provides a transition programme from adolescence to adulthood.

## Graphical abstract

Severe neutropenia can result from decreased production of neutrophil precursors in the bone marrow, as in the case of severe congenital neutropenia, or from increased utilization of neutrophils or their accelerated destruction as for drug‐induced neutropenia or autoimmune neutropenia. Severe chronic neutropenia increases susceptibility to bacterial or fungal infections.

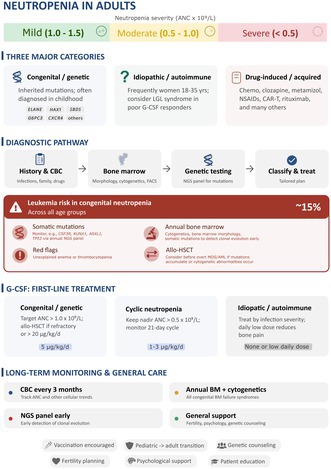

## INTRODUCTION

In a haematology outpatient clinic, neutropenia is a common reason for further evaluation. Most patients have acute or transient neutropenia related to treatment for underlying disorders or acute viral infections. Adult patients with neutropenia, defined as a blood absolute neutrophil count (ANC) of less than 1.5 × 10^9^/L, require special attention. Mild neutropenia (ANC 1.0–1.5 × 10^9^/L) often receives little attention unless it is part of a systemic illness such as rheumatoid arthritis [RA], systemic lupus erythematosus or other autoimmune disorders. An ANC of 0.5–1.0 × 10^9^/L, that is, moderate neutropenia, may be associated with symptoms such as fever and fatigue. Severe chronic neutropenia (SCN), defined as an ANC of less than 0.5 × 10^9^/L over the course of weeks or months, is much less common and more serious. In this article, we present our expert advice on the diagnosis and treatment of adults with SCN (see also Ref [^1^, ^2^, ^3^, ^4^, ^5^, ^6^, ^7^, ^8^, ^9^, ^10^, ^11^]).

Severe chronic neutropenia in adults is usually encountered in one of the three situations:

- Routine blood count of ANC <0.5 × 10^9^, with or without other haematological findings;
- Recurrent fevers, pharyngitis, sinusitis, otitis, stomatitis or respiratory infections which may be slow to resolve;
- Family history of SCN caused by a germline mutation.

It should be noted that some individuals of African and Middle Eastern descent display normal ANCs in the range from 0.5 to 1.5 × 10^9^/L and less frequently even lower.^9^ The term atypical chemokine receptor (ACKR1/DARC)‐associated neutropenia (ADAN), instead of ethnic neutropenia, is now used to emphasize the genetic rather than the ethnic basis of this entity.

Cyclic neutropenia (CyN) is characterized by oscillating numbers of blood neutrophils, monocytes, platelets, reticulocytes and lymphocytes, usually with a 21‐day periodicity. Mutations in *ELANE* (Neutrophil Elastase) can cause cyclic neutropenia and, although there is some overlap in *ELANE* mutations with CN, genotype–phenotype correlations indicate that there are mutations that are more likely to be associated with CyN. When neutrophil counts are at the lowest point in the nadir, patients regularly have painful mouth ulcers, respiratory symptoms, cellulitis, abscesses and severe infections.^10^

## NEUTROPENIA IN ADULTS CAUSED BY GERMLINE MUTATIONS

In adult patients with chronic neutropenia, an underlying inborn genetic disorder is possible, and conditions diagnosed in early childhood are increasingly important also in adult haematology. Due to early initiation of treatment for congenital neutropenia (CN), patients experience a better life expectancy and reach adulthood. These patients usually have good knowledge of their disorder and are familiar with a history of diagnostic procedures, germline mutations, risk of infectious episodes or haematological malignancies, necessity of therapy and surveillance. Coordinated transition from paediatric to adult haematology is crucial for the continuation of surveillance in adult CN patients.

However, congenital conditions may sometimes be un‐diagnosed, and patients may be referred to an adult haematologist for the first time. The most common type of inborn neutropenia diagnosed in adulthood is cyclic neutropenia (CyN), caused by a mutation in the *ELANE* gene.^10^ There are different possible reasons why CyN may not have been diagnosed during childhood. In most cases, the rise in neutrophil counts following the nadir is misinterpreted as spontaneous recovery, and further monitoring is not performed. In other patients with cyclic neutropenia, neutrophil counts at their nadir remain within a range in which infections occur only sporadically or rarely (above 0.5 × 10^9^/L). Sometimes, it is simply an inadequacy of the healthcare system that leads to delayed diagnosis.

**FIGURE 1:**
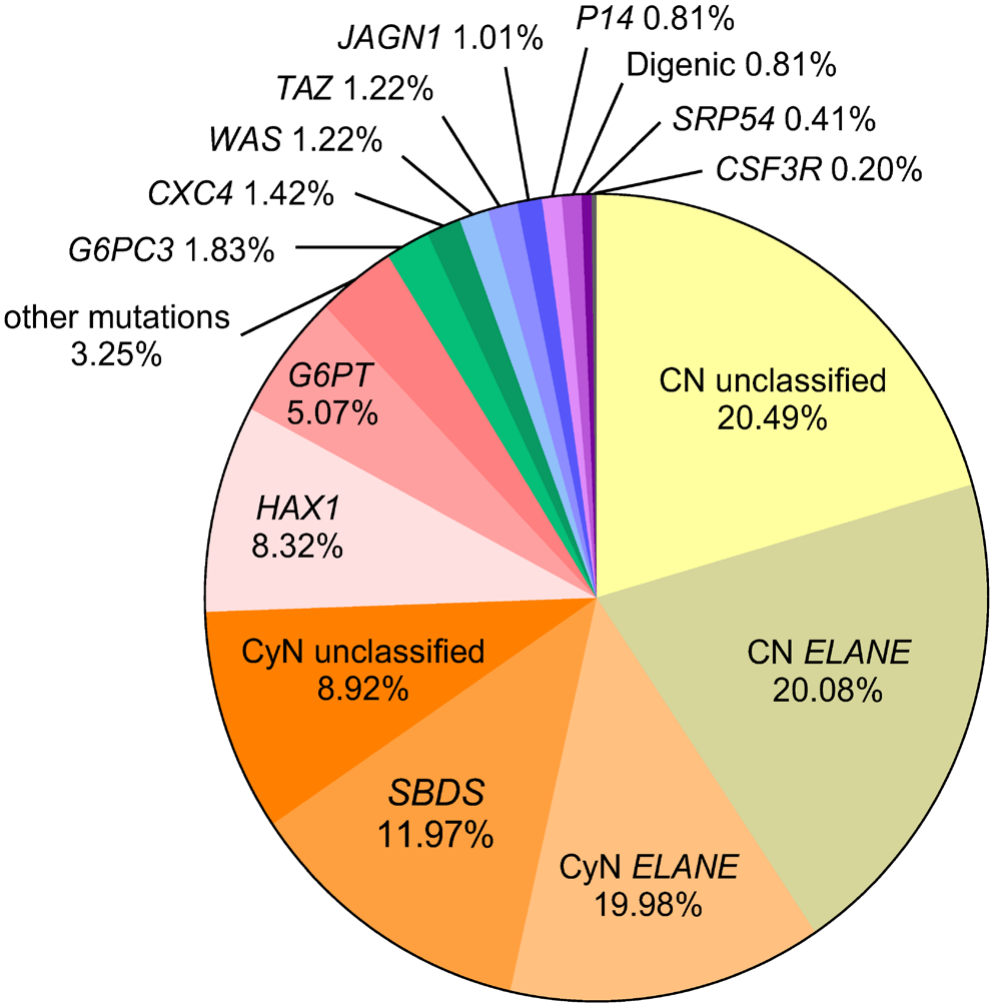
Genotype distribution among patients with inherited neutropenia over the age of 18 (*n* = 493, 09/2025).

In the European branch of the Severe Chronic Neutropenia International Registry (SCNIR‐Europe, https://severe‐chronic‐neutropenia.org/de), we currently oversee 458 patients with congenital persistent and cyclic neutropenia who are older than 18 years. The most frequent neutropenia causing germline mutations are identified in the *ELANE* gene (163 pts) with cyclic or persistent neutropenia. Other genes involved are *HAX1* (41 pts), *SBDS* (59 pts), *G6PT* (25 pts), *G6PC3* (9 pts), *CXCR4* (7 pts), *TAZ* (6 pts), *JAGN1* (5 pts) or others (e.g. *WAS*, *LAMTOR2*, *CSF3R* (together 33 pts)). For some patients with suspected congenital neutropenia, no genetic cause of neutropenia was identified yet (unclassified neutropenia, 145 pts) (Figure 1).

Congenital neutropenia may be caused by mutations leading to isolated severe neutropenia or neutropenia in combination with non‐haematological organ involvements, including the heart (*G6PC3*, *TAZ, SBDS*), central nervous system (*HAX, SBDS, CLPB*), urogenital system (*G6PC3*), skeleton (*SBDS, JAGN1*), exocrine pancreas (*SBDS, SRP54*), liver (glycogen storage; *SLC37A4*), skin (*SBDS* ^*14*^, *G6PC3, AP3B1, LAMTOR, LYST, RAB27A*) and metabolic pathways (*SLC37A4, TAZ, CLPB*).^10^, ^11^

The involvement of the reproductive organs and fertility issues in HAX‐1 associated CN have only recently been described, as the number of patients in puberty or post‐puberty has increased, making statistical analysis feasible. These issues should ideally be addressed prior to family planning. Because current evidence suggests that female fertility is particularly at risk, and young women are already entering menopause shortly after puberty due to ovarian insufficiency, early consultation with gynaecologists is strongly recommended.

Shwachman–Diamond syndrome (SDS) is a rare ribosomal disorder primarily characterized by exocrine pancreatic insufficiency and bone marrow failure with neutropenia representing the most frequent haematological manifestation.^12^ In addition, there are variable organ manifestations, particularly affecting the skeletal system as well as a variant degree of neurodevelopmental delay. SDS is caused by compound heterozygous mutations in the SBDS gene.^12^, ^13^ The disease is commonly diagnosed in early childhood prompted by failure to thrive secondary to exocrine pancreatic insufficiency; however, patients with milder phenotypes may remain undiagnosed until adulthood. A diagnosis of SDS should be considered in adults with neutropenia presenting with short statue, concomitant thrombocytopenia and/or anaemia, and characteristic cytogenetic abnormalities including isochromosome 7 or deletion 20q in the bone marrow as observed in SDS patients. In cases of severe neutropenia and/or recurrent bacterial infection, treatment with G‐CSF may be considered. Studies have indicated that G‐CSF therapy does not increase the risk of developing MDS or leukaemia. The clinical manifestation of SDS is very variable. While some patients are able to lead largely independent lives, a subset remains significantly affected, exhibiting limited resilience and an inability to sustain the demands of a regular work routine or achieve full autonomy in daily life.

As in patients with congenital neutropenia, there is an increased lifelong risk of developing MDS and leukaemia in SDS. Acquired mutations in TP53 play a critical role in the leukaemogenesis in SDS patients. In case of malignant transformation, haematopoietic stem cell transplantation (HSCT) remains the only curative option.^12^ Due to the multisystemic nature of this disease, these patients require a multidisciplinary approach in patient care.

Since CN in general is a pre‐leukaemic condition with approximately 15% incidence of acute myeloid leukaemia (AML) or myelodysplastic syndrome (MDS), the question of how to detect malignant transformation at an early stage is the most important one for patients.^10^, ^13^, ^14^, ^15^, ^16^, ^17^, ^18^, ^19^, ^20^, ^21^, ^22^, ^23^, ^24^ Basic monitoring should, therefore, include regular complete blood counts (CBCs) at 3‐month intervals, annual bone marrow examination and cytogenetics are generally recommended but is immediately required, if ANC or other blood cells (red cells, platelets) drop without a reason and remain low. Long‐term surveillance includes regular (annual) monitoring of the acquisition of somatic leukaemia‐associated mutations in, for example, *CSF3R*, *RUNX1, ASXL1*, *TP53* and other leukaemia‐associated genes using panel next‐generation sequencing (NGS) to detect leukaemogenic transformation as early as possible. It is known that these mutations play a significant role in leukaemogenesis and were also frequently detected in CN and SDS patients who developed overt AML or MDS.^17^, ^18^, ^19^, ^20^, ^21^, ^22^, ^23^, ^24^

**FIGURE 2:**
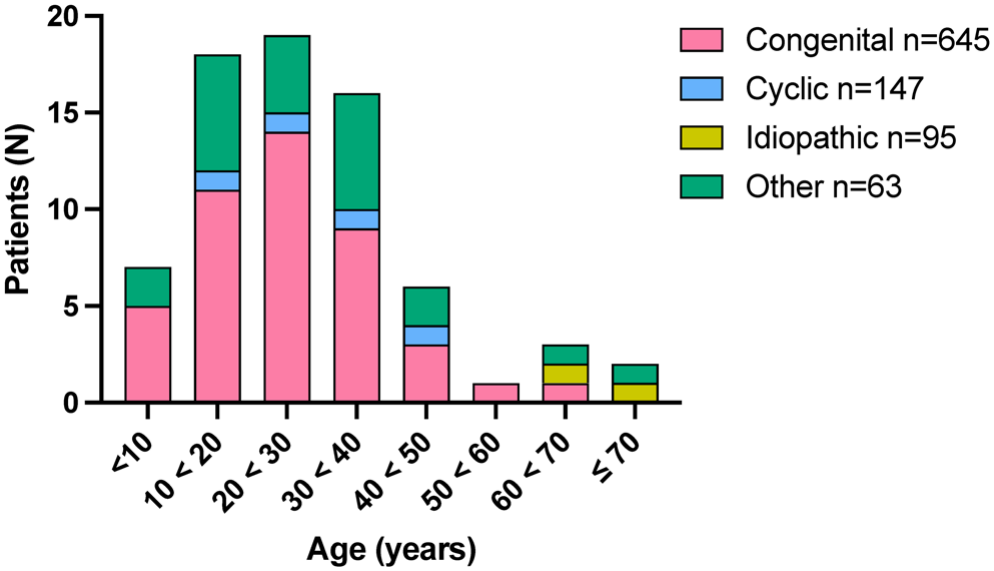
Number of CN patients over the age of 20 who developed MDS or AML (status: September 2025). For comparison, the number of CN patients below the age of 20 is also shown. AML, acute myeloid leukaemia; CN, congenital neutropenia; MDS, myelodysplastic syndrome.

Based on the data from the SCNIR‐Europe, an increased risk of MDS and AML transformation can be expected in all age groups. Despite limited numbers of adult patients diagnosed with CN, at the age between 20 and 30 years, 15 CN patients developed AML, at the age between 30 and 40 years—nine patients, and older than 40 years—six patients (Figure 2). This is an important difference from previous healthy individuals, in whom detectable leukaemia‐associated somatic mutations, clonal haematopoiesis and leukaemias are rare in people under 40 years of age and only become more frequent after the age of 70.^24^ Therefore, adult haematologists must understand that patients with CN have an increased lifetime risk of clonal evolution to leukaemia. Clinicians should be aware of the indications for allogeneic HSCT (allo‐HSCT)^25^, ^26^ in patients with a high frequency of haematopoietic stem cells that acquire cooperating leukaemia‐associated somatic mutations (e.g. *CSF3R*, *RUNX1* and *ASXL1*), prior to overt MDS/AML.

In general, we recommend G‐CSF treatment either in CN or in CyN as beneficial therapy because it reduces the frequency and severity of infection and improves the quality of life (reduced use of antibiotics, hospitalization and infection‐related complications).^27^, ^28^, ^29^, ^30^, ^31^, ^32^ Patients with benign ethnic neutropenia do not require G‐CSF treatment.^9^, ^31^ In patients with G‐CSF refractory disease, or very high G‐CSF dose requirements (over 20 μg/kg/d), allo‐HSCT should be considered. This diagnosis is usually made in early childhood^25^, ^26^

## CHRONIC IDIOPATHIC NEUTROPENIA IN ADULT

This type of neutropenia is a distinct condition predominantly affecting young adult women ageing 18–35 years. The patients have no chromosomal abnormalities or other evidence of myelodysplasia. Marrow examinations show a spectrum of abnormalities, ranging from normal cellularity to selective hypoplasia of the neutrophilic series. In most cases, quantitative marrow studies show the ratio of immature to mature cells is increased, suggesting loss of cells during the maturation process, that is, ineffective granulopoiesis. Neutrophil antibodies including anti‐nuclear or anti‐mitochondrial antibodies are absent.

For most patients, the clinical course can be predicted from the level of blood neutrophils, marrow examination and prior history of fevers and infections. In general, patients with the lowest levels of blood and marrow neutrophils have the most frequent problems. Long‐term observations show, however, that some patients have very low blood neutrophil levels for long periods with few or no infections. Evolution to lymphoid malignancies is the greatest concern and should be addressed in long‐term surveillance. G‐CSF increases neutrophils in most patients and is a useful therapy for patients with recurrent fever and infections.

Since the required G‐CSF doses are usually very low in Idiopathic Neutropenia (IDN), most female patients complain of severe bone pain following G‐CSF injection and require pain medication for 1–3 days afterwards when G‐CSF is administered only once or twice a week. Administering a lower dose at shorter intervals (ideally a low daily dose) significantly reduces bone pain and is well tolerated. Adult patients with SCN, ANCs <0.5 × 10^9^ over several months and patients with any additional significant haematological abnormality, that is, anaemia, thrombocytopenia, lymphocytosis or abnormal granulocytes in the peripheral blood film, should definitely have a bone marrow (BM) and cytogenetic evaluation.

Annual BM and cytogenetics surveillance should be performed in all patients with congenital bone marrow failure syndromes independent of ANC and treatment with G‐CSF and all patients with undefined SCN (after extensive investigation) with G‐CSF treatment. It may also be considered in patients with decreasing ANC or additional changes in other blood cell counts (e.g. anaemia and thrombocytopenia) or erythrocyte indices. We also recommend a bone marrow examination prior to G‐CSF start or start of other therapies, especially immune therapies including steroids.

In general, any clinically significant persistent change in blood counts while on G‐CSF treatment is an indication to repeat bone marrow examination. Based on long‐term observational studies of the SCNIR, in contrast to CN patients, patients with idiopathic neutropenia who respond readily to G‐CSF with clear clinical benefit and no substantial changes in their blood counts or general health do not need repetitive or annual bone marrow examinations.

Adult patients with a careful medical history indicative of acquired neutropenia, that is, a healthy childhood without recurrent fevers or infections, may have an autoimmune disorder not recognizable through testing for anti‐neutrophil antibodies (ANAs).^33^, ^34^, ^35^ Their cytopenia is selective; other blood cell counts are normal or near normal. The results of the bone marrow morphology tests and other tests, including the anti‐nuclear (ANA) antibody (ANA) tests andanti‐neutrophil antibodies tests, are normal. In general, therapy should be conservative and expectant. Intravenous γ‐globulin may transiently increase neutrophils, but the therapy is expensive and relatively ineffective. The response to glucocorticoid therapy is unpredictable. Daily or alternate‐day G‐CSF is effective but should be reserved for patients with recurrent infections.

Conversely, because autoimmune disorders often affect specific organs or multiple organ system (e.g. thyroid diseases, inflammatory bowel disease, lupus erythematosus, RA including Felty's syndrome, Sjogren syndrome, systemic sclerosis), validated tests for autoantibodies against other tissues, such as the thyroid, gastric mucosa and liver, provide useful clues, and we always begin with a reflexive ANA test.^33^, ^34^, ^35^ We have found that patients with or without ANAs or antibodies of any other type influence the responsiveness to G‐CSF; both groups respond well.

One caveat, patients with RA often complain of increasing joint pain if given G‐CSF to treat their neutropenia. It is unclear whether G‐CSF and the higher neutrophil counts it stimulates will worsen arthritis, but for most RA patients, the symptoms dictate either very low dose or no G‐CSF treatment.

We recommend an individual approach for patients with chronic idiopathic neutropenia and the final decision regarding continuous G‐CSF therapy should be based on the history and severity of infections rather than the ANC and encourage the continuous use of G‐CSF in rare patients who present with frequent and/or severe infections.

Some physicians have presumed that suppressing the autoimmune disease with corticosteroids, methotrexate, ciclosporin or other immune modulating drugs (IMDs) will be more effective than G‐CSF alone.^36^ There are no conclusive studies in this regard but generally we recommend avoiding concomitant treatment with these agents simply on the presumption that the disease is caused by autoimmunity.

Peripheral blood or bone marrow analysis using fluorescence activated cell sorting (FACS) is particularly useful for identifying clonal abnormalities of myeloid or lymphoid cells. Identification of large granular lymphocytes (LGLs) is sufficiently subtle that it is often missed or misdiagnosed. In our experience, LGL syndrome looks like chronic idiopathic neutropenia except for modestly higher lymphocyte counts than most idiopathic neutropenia patients. The diagnosis may be suggested by careful examination of the blood film, but it is almost always confirmed by FACS analysis showing increased numbers of cluster of differentiation (CD)56^+^ or CD57^+^ cells. Another clue to this diagnosis may be a relatively poor response to G‐CSF. Through experience, we have learned that weekly methotrexate plus G‐CSF is far more effective than either alone in LGL leukaemia. Alternatively, ciclosporin is the most frequent alternative to methotrexate and probably equally effective. In our experience, patients with neutropenia and increased numbers of CD56^+^ or CD57^+^ cells in the blood may go for very long periods without evolution and with a stable response to treatment. Vigilance, however, is encouraged because some patients will evolve to chronic lymphocytic leukaemia or multiple myeloma. We have not seen myeloid malignancies in this patient population.

Adults with idiopathic or presumed autoimmune neutropenia have arthralgias, bone pain and headaches with about the same frequency as other patients treated long term with G‐CSF. These side effects can be largely avoided by treatment with the lowest dose of G‐CSF that keeps the neutrophil count around 1.0 × 10^9^, but not higher. Symptoms seem to be less the more frequent the G‐CSF is administered, that is, daily or alternate day is much preferred to weekly or ‘as needed’ treatment.

Adults with chronic idiopathic neutropenia are not expected to evolve to another autoimmune disorder or to develop MDS or AML. If patients require G‐CSF, they can be expected to stay on the same low dose of G‐CSF for many years. Through the SCNIR, we have records on 448 adults with idiopathic or autoimmune neutropenia treated with G‐CSF for 0.02–30.8 years without evolution or significant change in treatment. We have not observed that these patients are prone to non‐myeloid malignancies, although they have these conditions with about the same frequency as the other adult population. As in almost every aspect of clinical medicine, there are exceptions. To be a good physician for these patients is to always be on the lookout on their behalf.

## DRUG‐INDUCED NEUTROPENIA

Our basic understanding of drug‐induced neutropenia is limited, partly because of the unpredictable occurrence of cases, the myriad agents involved and the lack of good animal models to experimentally study these conditions. Symptomatic patients with drug‐induced neutropenia usually present with fever, myalgia and sore throat but usually no rash or evidence of allergy elsewhere.^37^, ^38^ There is a long list of drugs capable of inducing neutropenia, many analgesics and non‐sterodial anti‐inflammatory drugs (NSAIDs), antibiotics (beta‐lactams, cefipime, trimethoprim‐sulfametoxazole, sulfasalazine, vancomycin, rifampicin, fluconazole, ketoconazole), anti‐diuretics (furosemide, spironolactone), antiretroviral human immunodeficiency virus (HIV) therapy, anti‐thyroids (tiamazofe, metimazole), clozapine (olanzapine), deferiprone, dipyrone (metamizole), phenothiazines (alimemazine) and quinine/quinidine are reported to induce neutropenia.^39^ Clinical studies suggest that the rate of recovery can be roughly predicted from the degree of marrow hypoplasia present when neutropenia is discovered. In patients with sparse marrow neutrophils but normal‐appearing precursor cells (promyelocytes and myelocytes), neutrophils reappear in the blood approximately 4–7 days after the offending drug is stopped. Often an increase in the blood monocyte count heralds marrow recovery, and an ‘overshoot’ with marked neutrophilia follows. When early precursor cells are severely depleted, recovery may require considerably more time.

Symptomatic patients with drug‐induced neutropenia usually present with fever, myalgia and sore throat but usually no rash or evidence of allergy elsewhere. Blood examination shows few or absent neutrophils. Mild lymphopenia may be observed, but other cell counts are usually normal. A high level of suspicion and careful clinical history are critical to identifying the offending drug. The differential diagnosis includes acute viral infections, particularly infectious mononucleosis and infectious hepatitis, and acute bacterial sepsis. If other haematological abnormalities are also present, acute leukaemia and aplastic anaemia should be considered. Treatment usually consists of supportive care, including broad‐spectrum antibiotics for febrile patients. Haematopoietic growth factors such as G‐CSF or GM‐CSF may be beneficial, but their use in this setting has not been established in randomized trials. An alternative to discontinuing drugs such as clozapine is the addition of G‐CSF treatment, although it is usually preferable to discontinue the suspected offending agent.

## FEBRILE NEUTROPENIA AFTER CANCER CHEMOTHERAPY AND CANCER IMMUNOTHERAPY

One of the most common side effects of cancer chemotherapy is febrile neutropenia.^28^ This condition leads to infections and hospital admissions in the majority of cancer patients. Febrile neutropenia is a well‐known indication for treatment with G‐CSF and intravenous antibiotics. For example, a study of patients with small‐cell lung cancer found that the rate of febrile neutropenia events was 57% in the first cycle of cyclophosphamide, doxorubicin and etoposide chemotherapy, rising to 77% by the end of six cycles.^40^ G‐CSF treatment reduced the incidence of febrile neutropenia to 28% during the first cycle and to 40% overall over the six cycles of treatment. This key finding, that G‐CSF reduces the incidence of chemotherapy‐induced febrile neutropenia by approximately 50%, has been confirmed by many other studies in subsequent years.^28^

Neutropenia is a common and significant complication of chimeric antigen receptor (CAR)‐T‐cell therapy.^41^, ^42^ It typically develops shortly after treatment and may persist or recur. Clinically, neutropenia can present in three phases: (1) early (within approximately 3 weeks): nearly universal and chemotherapy‐driven; (2) prolonged (lasting over 30 days): occurs in a subset of patients and is associated with severe inflammation and poor marrow recovery; (3) late (lasting over 90 days): less common and with unclear mechanisms (possibly ongoing immune dysregulation). The main cause of neutropenia is the systemic inflammation associated with cytokine release syndrome (CRS) following CAR‐T‐cell infusion; wherein high cytokine levels (mostly Interleukin‐6 and Interferon‐gammaI) cause bone marrow suppression and impair haematopoiesis. Other contributing factors include prior intensive therapies, reduced bone marrow reserve and immune‐mediated suppression of the bone marrow, wherein an activated immune system may inhibit progenitor cells and alter the bone marrow niche. Infections, including viral reactivation (e.g. Cytomegalovirus) and bacterial infections, are also contributing factors.^43^

Treatment with rituximab can also cause a late‐onset neutropenia (LON).^44^, ^45^, ^46^ LON typically occurs at least 4 weeks after the last dose of rituximab, most commonly between 4 and 12 weeks, but sometimes it may take several months to appear. LON is often transient but can reach severe levels (neutrophils <500/μL). The pathophysiology of LON is multifactorial and not fully understood. Several mechanisms have been suggested: (1) immune dysregulation following B‐cell depletion, including the development of ANAs or T‐cell–mediated suppression; (2) altered cytokine homeostasis leading to impaired granulopoiesis; (3) bone marrow microenvironment changes affecting haematopoietic recovery; and (4) in some cases, expansion of large granular lymphocytes (LGLs), which contribute to neutrophil suppression.^44^, ^45^, ^46^

## QUALITY OF LIFE OF PATIENTS WITH SEVERE CHRONIC NEUTROPENIA

There is a significantly negative impact of a chronic severe neutropenia disorder on quality of life (QOL), fatigue, physical function, cognitive function and pain in adult patients when compared to controls. Michniacki et al. reported that adults had worse scores for QOL, depression and fatigue when compared to children. Adult and paediatric chronic severe neutropenia patients or their caregivers felt that their medical provider was compassionate, trustworthy and accessible.^47^ However, less than 50% of adult patients agreed that their clinician had excellent expertise in white blood cell disorders. Chronic neutropenia complexly affects QOL.^47^

## SUMMARY RECOMMENDATIONS

**FIGURE 3:**
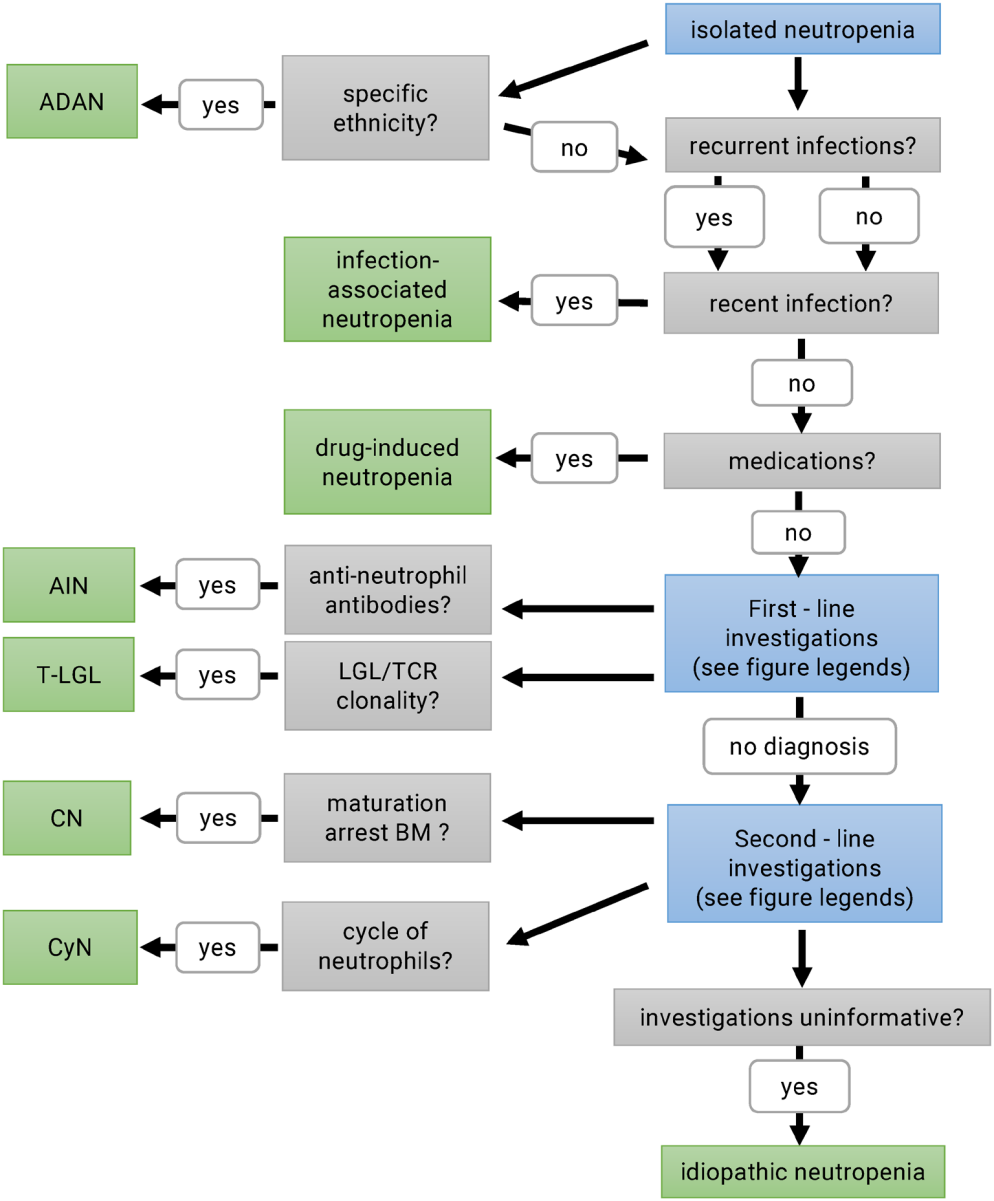
Flowchart for the basic evaluation of a patient with chronic neutropenia. First‐line investigations: Differential complete blood counts, peripheral blood film, liver and kidney function tests, immunoglobulin levels, vitamin B12, folic acid, C‐reactive protein, fluorescence activated cell sorting (FACS) of lymphocyte subsets, screening for hepatitis B and C, CMV, Epstein‐Barr‐Virus, parvovirus, human immunodeficiency virus; thyroid stimulating hormone, T3, T4, anti‐thyroid peroxidase, anti‐neutrophil antibodies, anti‐phospholipid and anti‐cardiolipin antibodies, FACS for LGL/Tcell‐receptor clonality in PB lymphocytes, serum ferritin, rheumatoid Factor, anti‐nuclear antibodies, extractable nuclear antigen, double‐stranded‐DNA and erythrocyte‐sedimentation‐rate. Second‐line investigations: CBCs in family members, copper, caeruloplasmin, anti‐tissue‐transglutaminase‐IgA, deamidated gliadin peptide antibodies, pancreatic isoamylase, serum electrophoresis, serum complement levels, next generation sequencing of somatic leukaemia‐associated mutations, bone marrow aspiration, bone marrow biopsy, cytogenetics. Cyclic neutropenia: Serial differential CBC 3x/week for 6 weeks. ADAN, ACKR1/DARC‐associated neutropenia; AIN, auto‐immune neutropenia; CN, congenital neutropenia; CyN, cyclic neutropenia; LGL, large granular lymphocytes.

Diagnostic procedures are shown in Figure 3.

For patients progressing from childhood to adulthood:

1. Careful handoff of the results and confirmed genetic diagnosis (e.g. *ELANE, HAX1* or many other mutations), phenotype (CN vs. CyN), history of infections and bone marrow findings.
2. Provide records of illnesses, treatment records and plan: G‐CSF regimen (dose, adjustments, ANC targets), history of prophylactic antibiotics/anti‐fungals and vaccination records.

For all patients:

1 G‐CSF treatment is beneficial for most adult patients with SCN and recurrent infections. It reduces the frequency and severity of infection and improves the quality of life (reduction of antibiotics, hospitalization and infection‐related complications).
2 In established CN without previous treatment, we recommend initiating G‐CSF at 5 μg/kg/day. In CyN and most patients with idiopathic neutropenia, it is prudent to begin with a lower dose of G‐CSF, for example, 1–3 mcg/kg/day or ≤3 μg/kg/day every other day. Dosage should be adjusted to avoid ANC nadir <0.5 × 10^9^/L CyN and ANC results <1.0 × 10^9^/L in other patients. Other treatments, for example, haematopoietic growth factors, corticosteroids or immunosuppressive agents, should be reserved for special circumstances and are generally not our recommendation for the treatment of SCN.
3 Documentation of response to treatment and complications is very important including hospitalizations, severe infections, abnormal bone marrow cytogenetics and any clonal abnormalities. CBC initially at diagnosis and every 1–3 months depending on stability and then about 3–4 times per year. When complications occur or with uncertainties, consultation with physicians experienced in the care of these patients is indicated.
4 An annual bone marrow examination with cytogenetic analysis is recommended for patients with congenital neutropenia, as well as for those with changes in blood counts or general health that suggest haematopoietic evolution to leukemia. Increasingly, NGS monitoring for somatic leukaemia‐associated mutations (*CSF3R, RUNX1, TET2, ASXL1*, etc.) is available and indicated to establish leukaemic evolution.
5 Emphasize patient education: adolescents/young adults need to understand their disease including inheritance patterns, treatment rationale and signs of infection.
6 Genetic counselling is very important for siblings, children and family planning.
7 Fertility and pregnancy counselling should address reproductive health, pregnancy planning and risks associated with neutropenia in pregnancy.
8 Psychological support is very important as patients struggle with the effects of chronic disease, fatigue, frequent medical appointments and anxiety about leukaemia risk.
9 Other health considerations: Neutropenia does not limit the efficacy or benefit of vaccinations. Routine vaccinations should be encouraged for most patients.^48^

## AUTHOR CONTRIBUTIONS

**Karl Welte:** Conceptualization; investigation; writing – original draft; writing – review and editing; data curation. **Julia Skokowa:** Investigation; data curation. **David Dale:** Conceptualization; investigation; writing – original draft; data curation. **Cornelia Zeidler:** Investigation; data curation. **Audrey Anna Bolyard:** Investigation; data curation. **Sabine Mellor‐Heineke:** Investigation; data curation.

## FUNDING INFORMATION

Funding was provided by the Madeleine Schickedanz‐Kinderkrebs‐Stiftung e.V., Jose Carreras Leukämie‐Stiftung and National Instituts of Health USA grant number 5R24AI162637.

## CONFLICT OF INTEREST STATEMENT

The authors have no conflicts of interest to disclose.

## Data Availability

Data are available upon request. Data collection and analysis in the framework of the SCNIR were approved by the ethics committees of the University Hospital Tübingen and the Hannover Medical School. Informed consent was obtained from all study participants.
